# Supplementary material for: Virological characteristics of the SARS-CoV-2 XBB variant derived from recombination of two Omicron subvariants
Source: Nat Commun. 2023 May 16;14:2800. doi: 10.1038/s41467-023-38435-3 (PMC10187524; doi:10.1038/s41467-023-38435-3)
Supplement: Supplementary file 1 — Supplementary Information (modified) [file 41467_2023_38435_MOESM1_ESM.pdf]

Supplementary Table 1. Estimated relative Re values of XBB-related lineages in India

PANGO lineage	Posterior mean	Posterior 2.5 percentile	Posterior 97.5 percentile	R-hat value	Effective sampling size (ESS_bulk)	Effective sampling size (ess_tail)
BA.2.10	0.99	0.97	1.01	1.002	3608.23	2989.56
BA.2.75	1.15	1.15	1.16	1.002	2225.09	2445.14
BA.5	1.08	1.08	1.09	1.002	2224.21	2557.34
BJ.1	1.22	1.20	1.25	1.000	2771.17	2785.13
BM.1	1.22	1.19	1.25	1.002	2678.88	2575.86
BM.1.1	1.31	1.28	1.35	1.001	3339.73	2797.80
BM.1.1.1	1.26	1.23	1.29	1.000	2905.66	2877.07
XBB	1.51	1.47	1.54	1.002	2389.68	2667.18
XBB.1	1.65	1.59	1.72	1.001	2741.92	2177.44

The R_e of BA.2 is set at 1.

Supplementary Table 2. Estimated lineage frequencies for BQ.1, XBB, and other lineages as of November 15, 2022 in each country

Country	BQ.1 lineage	XBB lineage	Other lineages
Australia	0.25	0.07	0.68
Austria	0.23	0.16	0.61
Bangladesh	0.00	1.00	0.00
Belgium	0.57	0.03	0.40
Brazil	0.68	0.00	0.32
Brunei	0.00	0.46	0.54
Canada	0.34	0.01	0.65
Chile	0.23	0.00	0.77
Croatia	0.25	0.00	0.75
Czech Republic	0.21	0.00	0.79
Denmark	0.40	0.04	0.56
Dominican Republic	0.22	0.73	0.05
Ecuador	0.87	0.00	0.13
Finland	0.24	0.16	0.60
France	0.73	0.02	0.25
Germany	0.22	0.01	0.78
Hong Kong	0.68	0.00	0.32
Iceland	0.72	0.00	0.28
India	0.00	0.91	0.09
Indonesia	0.29	0.52	0.19
Ireland	0.71	0.04	0.26
Israel	0.58	0.05	0.37
Italy	0.42	0.03	0.55
Japan	0.09	0.01	0.89
Kenya	0.89	0.00	0.11
Luxembourg	0.44	0.00	0.56
Malaysia	0.13	0.64	0.23
Mexico	0.44	0.00	0.56
Netherlands	0.43	0.08	0.49
Nigeria	1.00	0.00	0.00
Norway	0.65	0.07	0.28
Peru	0.33	0.44	0.23
Portugal	0.72	0.00	0.28
Puerto Rico	0.41	0.00	0.59
Reunion	0.66	0.00	0.34
Singapore	0.28	0.58	0.13
South Africa	0.16	0.00	0.84
South Korea	0.07	0.03	0.90
Spain	0.79	0.00	0.21
Sweden	0.45	0.07	0.48
Switzerland	0.46	0.06	0.49
Turkey	0.51	0.00	0.49
UK	0.58	0.04	0.38
USA	0.52	0.04	0.44
Costa Rica	0.00	0.00	1.00
Estonia	0.00	0.00	1.00
Greece	0.18	0.00	0.82
New Zealand	0.00	0.00	1.00
Philippines	0.00	0.87	0.13
Poland	0.09	0.00	0.91
Romania	0.32	0.00	0.68
Russia	0.00	0.00	1.00
Slovakia	0.00	0.00	1.00
Slovenia	0.17	0.00	0.83
Thailand	0.00	0.00	1.00
Vietnam	0.00	0.00	1.00

Supplementary Table 3. Estimated relative Re values of viral lineages by a hierarchical Bayesian model

Country	PANGO lineage	Posterior mean	Posterior 2.5 percentile	Posterior 97.5 percentile	R-hat value	Effective sampling size (ESS_bulk)	Effective sampling size (ess_tail)
Global average	BA.2.75	1.11	1.08	1.15	1.000	9677.27	4427.85
Global average	BQ.1	1.23	1.17	1.31	1.001	10792.68	5386.52
Global average	BQ.1.1	1.24	1.19	1.30	1.000	9884.49	4645.09
Global average	XBB	1.24	1.16	1.32	1.000	9616.06	5271.52
Global average	XBB.1	1.26	1.18	1.35	1.002	9487.80	4744.15
Australia	BA.2.75	1.10	1.10	1.11	1.000	13234.85	5442.32
Australia	BQ.1	1.16	1.15	1.17	1.001	11666.50	5521.52
Australia	BQ.1.1	1.20	1.18	1.21	1.001	13346.27	5673.41
Australia	XBB	1.14	1.12	1.15	1.002	10704.63	4997.43
Australia	XBB.1	1.15	1.13	1.16	1.003	10633.35	4773.28
Austria	BA.2.75	1.06	1.06	1.07	1.000	12124.96	5274.22
Austria	BQ.1	1.14	1.12	1.17	1.000	8124.21	4025.41
Austria	BQ.1.1	1.19	1.17	1.22	1.001	10282.15	5349.60
Austria	XBB	1.11	1.09	1.13	1.001	11703.04	5256.00
Austria	XBB.1	1.17	1.15	1.19	1.000	11561.46	5598.81
Denmark	BA.2.75	1.15	1.14	1.15	1.001	12052.64	5283.37
Denmark	BQ.1	1.17	1.16	1.18	1.001	12065.65	6237.99
Denmark	BQ.1.1	1.20	1.20	1.21	1.001	11679.24	6235.03
Denmark	XBB	1.22	1.19	1.26	1.001	12947.78	5180.74
Denmark	XBB.1	1.24	1.21	1.26	1.000	12052.24	5458.20
India	BA.2.75	1.10	1.09	1.11	1.000	9526.44	6804.82
India	BQ.1	1.34	1.20	1.53	1.000	9714.69	5731.72
India	BQ.1.1	1.24	1.07	1.45	1.000	9405.90	5061.28
India	XBB	1.32	1.30	1.34	1.000	8996.46	5522.31
India	XBB.1	1.41	1.37	1.45	1.000	10043.71	6121.81
Indonesia	BA.2.75	1.20	1.18	1.22	1.001	9931.69	6160.76
Indonesia	BQ.1	1.30	1.27	1.33	1.000	9265.32	5540.56
Indonesia	BQ.1.1	1.33	1.29	1.37	1.000	9552.47	6321.79
Indonesia	XBB	1.37	1.34	1.41	1.001	9545.41	6072.15
Indonesia	XBB.1	1.47	1.41	1.53	1.000	8737.60	5634.04
Israel	BA.2.75	1.09	1.08	1.10	1.000	10514.24	5724.24
Israel	BQ.1	1.18	1.17	1.19	1.001	12552.21	5673.30
Israel	BQ.1.1	1.22	1.21	1.23	1.000	10533.28	5583.56
Israel	XBB	1.18	1.15	1.21	1.000	12286.08	5845.43
Israel	XBB.1	1.19	1.17	1.22	1.001	10660.00	5728.63
Malaysia	BA.2.75	1.16	1.14	1.17	1.001	11277.18	5791.44
Malaysia	BQ.1	1.31	1.24	1.38	1.000	12210.94	6088.66
Malaysia	BQ.1.1	1.28	1.22	1.34	1.000	10594.23	5222.47
Malaysia	XBB	1.32	1.25	1.39	1.000	10526.66	5753.88
Malaysia	XBB.1	1.32	1.29	1.36	1.001	10614.16	5882.52
Peru	BA.2.75	1.14	1.11	1.18	1.000	15433.90	5559.18
Peru	BQ.1	1.37	1.29	1.47	1.002	9942.90	4933.82
Peru	BQ.1.1	1.30	1.25	1.36	1.000	10699.05	4468.45
Peru	XBB	1.53	1.41	1.69	1.000	9716.08	5304.72
Peru	XBB.1	1.27	0.99	1.61	1.000	9638.07	5021.15
Singapore	BA.2.75	1.06	1.05	1.07	1.000	10766.37	5609.37
Singapore	BQ.1	1.35	1.30	1.40	1.000	11801.21	6201.65
Singapore	BQ.1.1	1.37	1.32	1.43	1.000	10612.00	5357.87
Singapore	XBB	1.17	1.16	1.19	1.001	9735.23	5638.55
Singapore	XBB.1	1.18	1.17	1.19	1.001	8608.59	5687.14
United Kingdom	BA.2.75	1.13	1.12	1.13	1.001	10763.75	5108.79
United Kingdom	BQ.1	1.16	1.16	1.17	1.000	10183.40	6123.03
United Kingdom	BQ.1.1	1.20	1.19	1.20	1.002	10774.76	6190.40
United Kingdom	XBB	1.21	1.19	1.23	1.000	14035.02	5394.95
United Kingdom	XBB.1	1.32	1.29	1.35	1.001	13168.62	5858.59
USA	BA.2.75	1.09	1.09	1.09	1.001	15409.28	5383.66
USA	BQ.1	1.18	1.18	1.19	1.001	12198.48	4887.69
USA	BQ.1.1	1.22	1.21	1.22	1.001	12040.45	4931.51
USA	XBB	1.20	1.18	1.21	1.001	14760.39	5823.37
USA	XBB.1	1.27	1.25	1.29	1.000	11720.44	5365.18

The R_e value of BA.5 is set at 1.

Supplementary Table 4. Cryo-EM data collection, refinement and validation statistics

	SARS-CoV-2 XBB.1 spike		SARS-CoV-2 XBB.1 spike-ACE2		
**Data collection and processing**	**closed-1**	**closed-2**	**1-up**	**2-up**	**RBD-ACE2 interface**
EMDB ID	EMD-35622	EMD-35623	EMD-35624	EMD-35625	EMD-35626
PDB ID	8IOS	8IOT	8IOU	-	8IOV
Microscope	Krios G4		Krios G4		
Camera	Gatan K3		Gatan K3		
energy filter	Gatan Biocontinuum		Gatan Biocontinuum		
slit width	20		20		
Magnification	130,000		130,000		
Recording mode	counting		counting		
Voltage (kV)	300		300		
Electron exposure (e–/Å^2)	50.4 or 56.4		50.4 or 56.4		
Exposure time (s)	1.5		1.5		
Number of raw frames	50		50		
Defocus range (μm)	-0.8 to -1.8		-0.8 to -1.8		
Pixel size (Å)	0.67		0.67		
Initial particle images (no.)	1,966,806		1,630,799		
Final particle images (no.)	104,900	160,906	41,071	67,132	153,882
Symmetry imposed	C3	C3	C1	C1	C1
Map resolution (Å)					
FSC 0.143	2.50	2.51	3.18	2.99	3.29
**Refinement**					
Initial model used (PDB code)	8GS6	8GS6	8GS6, 7XB0	-	8GS6, 7XB0
Model composition					
Protein residues	3189	3063	3573	-	791
Ligands	NAG:54	NAG:48	BMA:2, NAG:56, MAN:2		BMA:2, NAG:11, MAN:2
Map CC	0.87	0.88	0.86	-	0.82
R.m.s. deviations					
Bond lengths (Å)	0.002	0.002	0.003	-	0.002
Bond angles (°)	0.488	0.524	0.515		0.530
Validation					
MolProbity score	1.33	1.64	1.50		1.57
Clashscore	4.04	5.18	5.19	-	5.90
Rotamer outliers (%)	0.00	0.00	0.00		0.00
Ramachandran plot					
Favored (%)	97.24	94.63	96.51		96.32
Allowed (%)	2.76	5.37	3.49	-	3.68
Outliers (%)	0.00	0.00	0.00		0.00

Supplementary Table 5. Human sera used in this study

Vaccine sera

Donor ID	Sex	Age	vaccine (1st vaccination)	1st vaccination (YYYY-MM-DD)	vaccine (2nd vaccination)	2nd vaccination (YYYY-MM-DD)	vaccine (3rd vaccination)	3rd vaccination (YYYY-MM-DD)	vaccine (4th vaccination)	4th vaccination (YYYY-MM-DD)	vaccine (5th vaccination)	5th vaccination (YYYY-MM-DD)	Date of sampling (YYYY-MM-DD)	Prior infection?
4	Female	43	BNT162b2	2021-05-14	BNT162b2	2021-06-04	BNT162b2	2022-01-28	BNT162b2	2022-08-01			2022-08-22	No
8	Female	56	BNT162b2	2021-05-14	BNT162b2	2021-06-04	BNT162b2	2022-01-28	BNT162b2	2022-08-05			2022-08-26	No
14	Female	43	BNT162b2	2021-05-14	BNT162b2	2021-06-04	BNT162b2	2022-01-28	BNT162b2	2022-08-05			2022-08-26	No
28	Female	42	BNT162b2	2021-04-06	BNT162b2	2021-04-28	BNT162b2	2022-01-22	BNT162b2	2022-07-30			2022-08-23	No
62	Male	46	BNT162b2	2021-05-14	BNT162b2	2021-06-10	BNT162b2	2022-01-28	BNT162b2	2022-08-05			2022-08-26	No
63	Female	49	BNT162b2	2021-05-12	BNT162b2	2021-05-31	BNT162b2	2022-02-05	BNT162b2	2022-08-02			2022-08-25	No
67	Male	38	BNT162b2	2021-05-11	BNT162b2	2021-06-01	BNT162b2	2022-02-01	BNT162b2	2022-08-03			2022-08-24	No
70	Female	48	BNT162b2	2021-05-12	BNT162b2	2021-06-02	BNT162b2	2022-01-22	BNT162b2	2022-08-04			2022-08-25	No
90	Male	45	BNT162b2	2021-05-11	BNT162b2	2021-06-02	BNT162b2	2022-01-25	BNT162b2	2022-08-02			2022-08-24	No
92	Female	30	BNT162b2	2021-05-20	BNT162b2	2021-06-10	BNT162b2	2022-01-26	BNT162b2	2022-08-04			2022-08-26	No
94	Male	36	BNT162b2	2021-05-11	BNT162b2	2021-06-01	BNT162b2	2022-02-02	BNT162b2	2022-08-05			2022-08-26	No
97	Female	43	BNT162b2	2021-05-12	BNT162b2	2021-06-04	BNT162b2	2022-01-26	BNT162b2	2022-07-21			2022-08-19	No
100	Male	32	BNT162b2	2021-05-18	BNT162b2	2021-06-08	BNT162b2	2022-01-22	BNT162b2	2022-07-17			2022-08-18	No
103	Male	35	BNT162b2	2021-03-17	BNT162b2	2021-04-07	BNT162b2	2022-01-25	BNT162b2	2022-08-02			2022-08-26	No
122	Female	45	BNT162b2	2021-05-20	BNT162b2	2021-06-11	BNT162b2	2022-02-16	BNT162b2	2022-08-01			2022-08-22	No
115117	Male	65	BNT162b2	2021-08-01	BNT162b2	2021-08-22	BNT162b2	2022-03-25	BNT162b2 (BA.1)	2022-10-01			2022-10-22	No
210718	Female	55	NA	NA	NA	NA	NA	2022-04-10	BNT162b2 (BA.1)	2022-10-02			2022-10-23	No
331843	Male	58	NA	2021-08-22	NA	2021-10-03	BNT162b2	2022-04-23	BNT162b2 (BA.1)	2022-10-03			2022-10-23	No
2362037	Male	57	NA	NA	NA	2021-08-29	BNT162b2	2022-04-07	BNT162b2 (BA.1)	2022-09-29			2022-10-21	No
108127	Female	57	BNT162b2	2021-08-07	BNT162b2	2021-09-08	BNT162b2	2022-04-07	BNT162b2 (BA.1)	2022-09-30			2022-10-21	No
190497	Female	52	BNT162b2	2021-08-30	BNT162b2	2021-09-21	mRNA-1273	2022-03-28	BNT162b2 (BA.1)	2022-10-01			2022-10-23	No
1437	Female	73	BNT162b2	2021-07-31	BNT162b2	2021-09-08	BNT162b2	2022-04-24	BNT162b2 (BA.1)	2022-09-28			2022-10-22	No
247029	Female	50	NA	2021-09-21	NA	2021-10-20	BNT162b2	2022-04-28	BNT162b2 (BA.1)	2022-09-28			2022-10-21	No
2382760	Female	30	mRNA-1273	2021-07-08	BNT162b2	2021-10-10	BNT162b2	2022-04-21	BNT162b2 (BA.1)	2022-09-28			2022-10-17	No
129061	Male	42	NA	NA	NA	NA	BNT162b2	2022-04-14	BNT162b2 (BA.1)	2022-10-01			2022-10-22	No
318009	Female	58	BNT162b2	2021-08-06	BNT162b2	2021-08-30	BNT162b2	2022-03-18	BNT162b2 (BA.1)	2022-09-29			2022-10-26	No
218082	Female	45	BNT162b2	2021-09-24	BNT162b2	2021-10-15	BNT162b2	2022-04-16	BNT162b2 (BA.1)	2022-09-30			2022-10-29	No
7371	Female	45	NA	NA	NA	NA	mRNA-1273	2022-04-23	BNT162b2 (BA.1)	2022-10-08			2022-10-30	No
145410	Male	71	BNT162b2	2021-07-23	BNT162b2	2021-08-23	BNT162b2	2022-03-18	BNT162b2 (BA.1)	2022-10-08			2022-10-29	No
2362083	Female	57	BNT162b2	2021-09-26	BNT162b2	2021-10-17	BNT162b2	2022-04-17	BNT162b2 (BA.1)	2022-10-08			2022-10-29	No
2420192	Female	36	BNT162b2	2021-03-08	BNT162b2	2021-03-29	BNT162b2	2021-12-10	BNT162b2 (BA.1)	2022-10-08			2022-10-29	No
149621	Female	80	BNT162b2	2021-06-29	BNT162b2	2021-07-28	BNT162b2	2022-03-04	BNT162b2 (BA.1)	2022-10-07			2022-10-28	No
309473	Female	57	NA	NA	NA	2021-09-17	NA	NA	BNT162b2 (BA.1)	2022-10-07			2022-11-01	No
2385248	Male	56	NA	NA	NA	NA	BNT162b2	2022-05-18	BNT162b2 (BA.1)	2022-10-06			2022-10-30	Yes (2021-08-14)
2362137	Male	63	NA	NA	NA	NA	mRNA-1273	2022-03-04	BNT162b2 (BA.1)	2022-10-06			2022-10-29	No
KS221117	Male	40	BNT162b2	2021-06-17	BNT162b2	2022-07-07	mRNA-1273	2022-03-28	BNT162b2 (BA.5)	2022-10-27			2022-11-17	No
216850	Female	59	BNT162b2	2021-09-04	BNT162b2	2021-09-25	BNT162b2	2022-04-07	BNT162b2 (BA.5)	2022-10-27			2022-11-21	No
226147	Male	49	BNT162b2	2021-09-21	BNT162b2	2021-10-20	BNT162b2	2022-05-21	BNT162b2 (BA.5)	2022-10-27			2022-11-21	No
2422938	Female	27	BNT162b2	2021-09-10	BNT162b2	2021-11-02	BNT162b2	2022-05-	BNT162b2 (BA.5)	2022-10-31			2022-11-22	No
2422916	Female	54	BNT162b2	2021-09-28	BNT162b2	2021-10-19	BNT162b2 (BA.5)	2022-10-31					2022-11-22	No
2422887	Male	32	BNT162b2	2021-08-23	mRNA-1273	2021-09-21	mRNA-1273	2022-03-28	BNT162b2 (BA.5)	2022-10-31			2022-11-22	Yes (2021-08-)
2404750	Female	51	BNT162b2	2021-07-23 or 2021-07-24	BNT162b2	2021-08-12	BNT162b2	2022-03-16	BNT162b2 (BA.5)	2022-10-31			2022-11-22	Yes (2022-07-08)
2363002	Male	52	BNT162b2	2021-08-27	BNT162b2	2021-09-17	BNT162b2	2022-04-09	BNT162b2 (BA.5)	2022-10-29			2022-11-23	No
1053	Male	86	BNT162b2	2021-05-17	NA	2021-06-07	NA	NA	NA	NA	BNT162b2 (BA.5)	2022-11-01	2022-11-24	No
2420132	Female	50	BNT162b2	2021-08-27	BNT162b2	2021-09-17	BNT162b2	2022-03-24	BNT162b2 (BA.5)	2022-10-28			2022-11-23	No
2423214	Male	77	BNT162b2	2021-03-	NA	2021-03-	NA	2022-03-29	NA	2022-07-05	BNT162b2 (BA.5)	2022-11-01	2022-11-24	No
2423218	Female	82	BNT162b2	2021-03-	NA	2021-03-	NA	2022-03-29	NA	2022-07-05	BNT162b2 (BA.5)	2022-11-01	2022-11-24	No
233746	Male	52	BNT162b2	2021-07-	NA	2021-07-	NA	2022-03-	BNT162b2 (BA.5)	2022-10-31			2022-11-24	No
NM221207	Female	54	BNT162b2	2021-06-26	BNT162b2	2021-07-17	BNT162b2	2022-02-05	BNT162b2 (BA.5)	2022-11-16			2022-12-07	No
2366112	Female	42	NA	2021-10-01	NA	2021-10-	NA	2022-05-01	BNT162b2 (BA.5)	2022-11-01			2022-11-25	No
14137	Female	41	BNT162b2	2021-09-10	BNT162b2	2021-10-01	BNT162b2	2022-04-16	BNT162b2 (BA.5)	2022-10-29			2022-11-27	No
2418539	Female	72	NA	NA	NA	NA	BNT162b2	2021-07-22	BNT162b2 (BA.5)	2022-11-06			2022-11-27	No
2420249	Male	47	BNT162b2	2021-09-05	BNT162b2	2021-09-26	BNT162b2	2022-04-10	BNT162b2 (BA.5)	2022-10-30			2022-11-27	No
2423718	Female	49	BNT162b2	2021-09-	BNT162b2	2022-01-	BNT162b2	2022-04-	BNT162b2 (BA.5)	2022-11-03			2022-11-27	No
319561	Male	18	BNT162b2	2021-09-21	BNT162b2	2021-10-12	BNT162b2	2022-04-15	BNT162b2 (BA.5)	2022-11-01			2022-11-27	No
2372043	Male	43	NA	NA	NA	NA	BNT162b2	2022-05-26	BNT162b2 (BA.5)	2022-11-01			2022-11-28	No

(Table S5, continued)

Breakthough infection sera												
SARS-CoV-2 infected	Donor ID	Sex	Age	Date of test (YYYY/MM/DD)	Date of sampling (YYYY/MM/DD)	Prior infection?	Prior vaccination?	Vaccine	1st vaccination (YYYY/MM/DD)	2nd vaccination (YYYY/MM/DD)	3rd vaccination (YYYY/MM/DD)	4th vaccination (YYYY/MM/DD)
BA.2	P378	Male	43	2022/03/28	2022/04/10	No	Yes	BNT162b2	2021/10/10	2021/10/31		
BA.2	P398	Male	48	2022/04/13	2022/04/30	No	Yes	BNT162b2	2021/09/18	2021/10/09	2022/04/09	
BA.2	P407	Male	29	2022/05/01	2022/05/12	No	Yes	mRNA-1273	2021/09/13	2021/10/11		
BA.2	P401	Male	35	2022/04/22	2022/05/05	No	Yes	BNT162b2	2021/09/09	2021/09/30		
BA.2	P412	Female	82	2022/05/04	2022/05/26	No	Yes	BNT162b2	2021/06/11	2021/07/09		
BA.2	6449	Male	43	2022/04/03	2022/04/23	No	Yes	BNT162b2	2021/08/13	2021/09/11		
BA.2	6355	Male	50	2022/04/02	2022/04/20	No	Yes	NA	2021/04/28	2021/05/19	2022/01/19	
BA.2	6547	Male	54	2022/04/06	2022/04/22	No	Yes	BNT162b2	2021/08/25	2021/09/15		
BA.2	7951	Female	71	2022/04/25	2022/05/12	No	Yes	Mix	2021/06/20 (BNT162b2)	2021/07/16 (BNT162b2)	2022/02/16 (mRNA-1273）	
BA.2	8645	Female	41	2022/05/07	2022/05/20	No	Yes	BNT162b2	2021/05/23	2021/06/13	2022/01/20	
BA.2	8682	Female	25	2022/05/08	2022/05/24	No	Yes	BNT162b2	2021/09/03	2021/09/27		
BA.2	5949	Male	24	2022/03/22	2022/05/22	No	Yes	mRNA-1273	2021/08/05	2021/09/02		
BA.2	8463	Male	84	2022/05/05	2022/05/23	No	Yes	Mix	2021/05/27 (BNT162b2)	2021/06/21 (BNT162b2)	2022/02/22 (mRNA-1273）	
BA.2	8796	Female	34	2022/05/10	2022/06/05	No	Yes	BNT162b2	2021/09/26	2021/10/17		
BA.5	P427	Female	49	2022/07/06	2022/07/25	No	Yes	NA	2021/07/30	2021/08/25	2022/03/18	
BA.5	P440	Male	25	2022/07/24	2022/08/07	No	Yes	BNT162b2	2021/11/24	2021/12/15		
BA.5	P439	Female	73	2022/07/23	2022/08/08	No	Yes	BNT162b2	2021/06/19	2021/07/20	2022/02/04 (mRNA-1273)	
BA.5	P451	Female	55	2022/07/29	2022/8/12	No	Yes	BNT162b2	2021/04/26	2021/05/20	2022/01/18	
BA.5	P456	Male	44	2022/08/04	2022/08/14	No	Yes	BNT162b2	2021/08/11	2021/09/01	2022/03/13 (mRNA-1273)	
BA.5	P455	Male	29	2022/08/03	2022/08/17	No	Yes	mRNA-1273	2021/07/26	2021/08/27	2022/04/18	
BA.5	P464	Male	63	2022/08/08	2022/08/19	No	Yes	BNT162b2	2021/08/08	2021/08/29	2022/04/07 (mRNA-1273)	
BA.5	9341	Male	56	2022/06/12	2022/06/30	No	Yes	BNT162b2	2021/08/10	2021/08/31	2022/03/18	
BA.5	9584	Male	55	2022/07/08	2022/07/25	No	Yes	BNT162b2	2021/07/14	2021/08/05	2022/03/22	
BA.5	11318	Female	51	2022/07/24	2022/08/05	No	Yes	BNT162b2	2021/09/01	2021/09/22	2022/05/19 (mRNA-1273)	
BA.5	23S-08	Male	25	2022/07/23	2022/08/08	No	Yes	BNT162b2	2021/04/27	2021/05/18	2022/01/11	
BA.5	11597	Female	41	2022/07/26	2022/08/08	No	Yes	BNT162b2	2021/04/30	2021/05/21	2022/01/11	
BA.5	10978	Female	46	2022/07/22	2022/08/11	No	Yes	BNT162b2	2021/08/27	2021/09/17	2022/05/15	
BA.5	10826	Male	63	2022/07/21	2022/08/11	No	Yes	BNT162b2	2021/07/27	2021/08/17	2022/03/04	2022/8/9 (mRNA-1273)
BA.5	11079	Female	65	2022/07/23	2022/08/11	No	Yes	mRNA-1273	2021/07/08	2021/08/05	2022/03/17	
BA.5	14847	Female	70	2022/08/13	2022/08/25	No	Yes	BNT162b2	2021/07/13	2021/08/20	2022/03/08 (mRNA-1273)	
BA.5	13180	Female	63	2022/08/04	2022/08/25	No	Yes	BNT162b2	2021/07/16	2021/08/06	2022/03/08 (mRNA-1273)	
BA.5	12912	Male	64	2022/08/02	2022/08/25	No	Yes	mRNA-1273	2021/09/02	2021/09/30	2021/04/01	
BA.5	14956	Female	33	2022/08/13	2022/08/28	No	Yes	BNT162b2	2021/09/06	2021/10/07		
BA.5	15707	Female	52	2022/08/16	2022/08/29	No	Yes	BNT162b2	2021/08/07	2021/08/28	2022/04/03	

NA, not applicable.

Supplementary Table 6. Primers used in this study

Primer name	Primer sequence (5'-to-3')	Purpose
Omicron universal Fw	cactatagggcgaattgggtaccatgtttgtgttcctggt	Preparation of S expression plasmid
BA2 Rv	agctccaccgcggtggcggccgctcaggtgtagtgcagtttca	Preparation of S expression plasmid
pC-S_BA.2_V83A_F	aggtttgacaaccctGCCctgccattcaatgat	Preparation of S expression plasmid
pC-S_BA.2_V83A_R	atcattgaatggcagGGCagggttgtcaaacct	Preparation of S expression plasmid
pC-S_BA.2_Y144del_F	ccattcctgGACgtctaccacaagaacaac	Preparation of S expression plasmid
pC-S_BA.2_Y144del_R	gttgttcttgtggtagacGTCcaggaatgg	Preparation of S expression plasmid
pC-S_BA.2_H146Q_F	ctgGACgtctactacCAGaagaacaacaagtcc	Preparation of S expression plasmid
pC-S_BA.2_H146Q_R	ggacttgttgttcttCTGgtagtagacGTCcag	Preparation of S expression plasmid
pC-S_BA.2_Q183E_F	gacttggagggcaagGAGggcaacttcaagaac	Preparation of S expression plasmid
pC-S_BA.2_Q183E_R	gttcttgaagttgccCTCcttgccctccaagtc	Preparation of S expression plasmid
pC-S_BA.2_G213E_F	acaccaatcAACCTGGAGAGGgacctgccacag	Preparation of S expression plasmid
pC-S_BA.2_G213E_R	ctgtggcaggtcCCTCTCCAGGTTgattggtgt	Preparation of S expression plasmid
pC-S_BA.2_G252V_F	tcctacctgacacctGTGgactcctcctctggc	Preparation of S expression plasmid
pC-S_BA.2_G252V_R	gccagaggaggagtcCACaggtgtcaggtagga	Preparation of S expression plasmid
pC-S_BA2_R346T_F	gtgttcaatgccaccACCtttgcctctgtctat	Preparation of S expression plasmid
pC-S_BA2_R346T_R	atagacagaggcaaaGGTggtggcattgaacac	Preparation of S expression plasmid
pC-S_BA.2_L368I_F	gctgactactctgtgATCtacaacTTCgccccc	Preparation of S expression plasmid
pC-S_BA.2_L368I_R	gggggcGAAgttgtaGATcacagagtagtcagc	Preparation of S expression plasmid
pC-S_BA.2_V445P_F	aagctggacagcaagCCCGGAggcaactacaac	Preparation of S expression plasmid
pC-S_BA.2_V445P_R	gttgtagttgccTCCGGGcttgctgtccagctt	Preparation of S expression plasmid
pC-S_BA.2_F486S_F	aatggagtggccggcAGCaactgttactttcca	Preparation of S expression plasmid
pC-S_BA.2_F486S_R	tggaaagtaacagttGCTgccggccactccatt	Preparation of S expression plasmid
pC-S_BA.2_F490S_F	ggcttcaactgttacAGCccactcagatcctat	Preparation of S expression plasmid
pC-S_BA.2_F490S_R	ataggatctgagtggGCTgtaacagttgaagcc	Preparation of S expression plasmid
pC-S_BA.2_Y144del/H146Q_F	ccattcctgGACgtctacCAGaagaacaacaagtcc	Preparation of S expression plasmid
pC-S_BA.2_Y144del/H146Q_R	ggacttgttgttcttCTGgtagacGTCcaggaatgg	Preparation of S expression plasmid
pC-S_BA.2_VG445-446PS_F	aagctggacagcaaCCCAGCggcaactacaac	Preparation of S expression plasmid
pC-S_BA.2_VG445-446PS_R	gttgtagttgccGCTGGGcttgctgtccagctt	Preparation of S expression plasmid
pC-S_BA.2_F486S/F490S/R493Q_F	aatggagtggccggcAGCaactgttacAGCccactcCAGtcctat	Preparation of S expression plasmid
pC-S_BA.2_F486S/F490S/R493Q_R	ataggaCTGgagtggGCTgtaacagttGCTgccggccactccat	Preparation of S expression plasmid

Supplementary Table 7. Summary of unexpected amino acid mutations detected in the working virus stocks

Original clinical isolate	Reference	Position in Reference (nt)	REF	ALT	Quality score	Depth in REF	Depth in ALT	Mutation type	ORF	Amino acid substitution
Delta (EPI_ISL_2378732)	NC_045512.2	17440	C	T	228	12	122	missense_variant	NSP13	P402S
Delta (EPI_ISL_2378732)	NC_045512.2	19010	A	G	228	15	137	missense_variant	NSP14	D324G
Delta (EPI_ISL_2378732)	NC_045512.2	27832	T	A	148	182	60	missense_variant	ORF7b	I26N
BA.2 (EPI_ISL_9595859)	NC_045512.2	no additional mutation								
BA.2.75 (EPI_ISL_13969765)	NC_045512.2	no additional mutation								
XBB.1 (EPI_ISL_15669344)	NC_045512.2	27682	T	C	139	169	66	missense_variant	ORF7a	Y97H

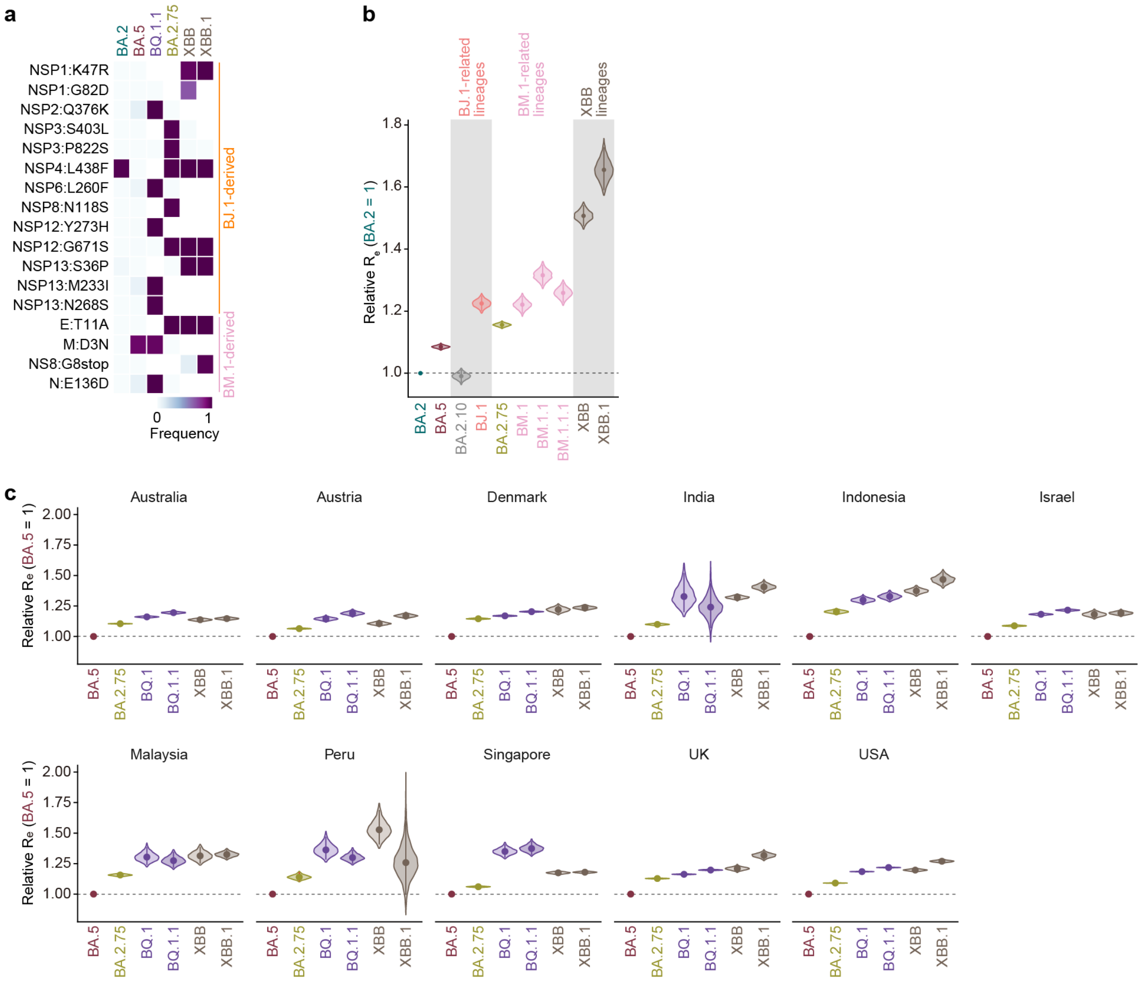

Supplementary Fig. 1. Phylogenetic and epidemic analyses of the XBB lineage

a, Amino acid differences in the non-S proteins of Omicron lineages.

b, Relative effective reproduction number (R_e) values for viral lineages in India, assuming a fixed generation time of 2.1 days, related to **Fig. 1e**. The R_e of BA.2 is set at 1. The posterior (violin), posterior mean (dot), and 95% Bayesian confidential interval (CI) (line) are shown.

c, Relative R_e values for viral lineages, assuming a fixed generation time of 2.1 days, related to **Fig. 1g**. The R_e value of BA.5 is set at 1. The posterior (violin), posterior mean (dot), and 95% Bayesian CI (line) are shown. R_e values for each country where BQ.1 and XBB lineages cocirculated are shown.

Source data are provided with this paper.

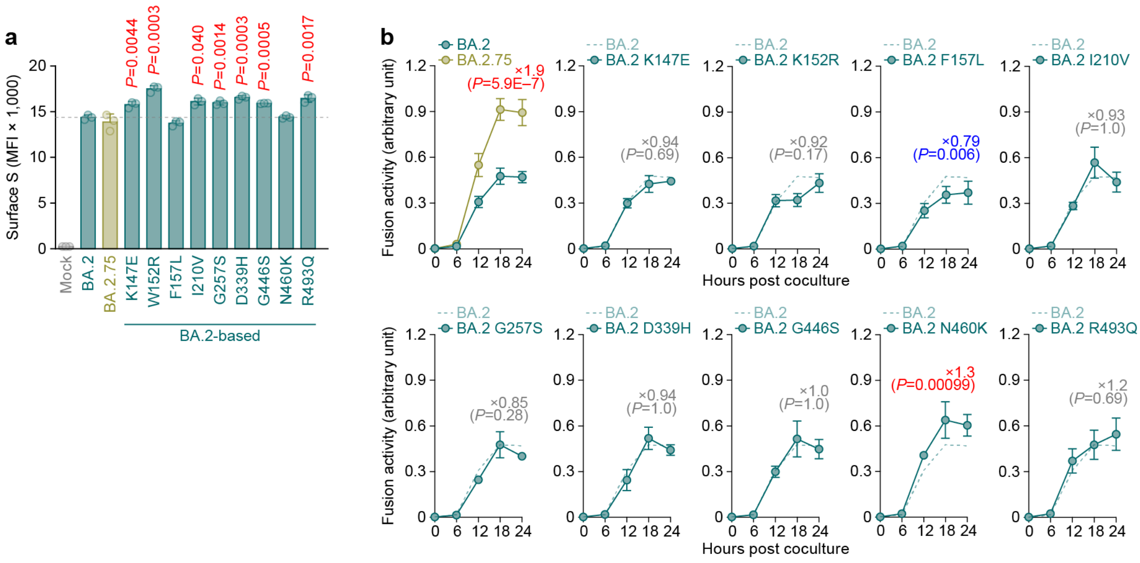

Supplementary Fig. 2. Fusogenicity of XBB.1 S

a,b, Spike (S)-based fusion assay. **a,** S protein expression on the cell surface. The summarized data are shown. **b,** S-based fusion assay in Calu-3 cells. The recorded fusion activity (arbitrary units) is shown. The dashed green line indicates the result of BA.2. The red number in each panel indicates the fold difference between BA.2 and the derivative tested (XBB.1 in the top left panel) at 24 h post coculture. Assays were performed in triplicate (**a**) or quadruplicate (**b**). The presented data are expressed as the average ± standard deviation (SD). In **a**, each dot indicates the result of an individual replicate, and the dashed horizontal lines indicate the value of BA.2. Statistically significant differences (*, $P < 0.05$) versus BA.2 were determined by two-sided Student's t tests, and red asterisks indicate increased values. In **b**, statistically significant differences versus BA.2 across timepoints were determined by multiple regression. The familywise error rates (FWERs) calculated using the Holm method are indicated in the figures. Source data are provided with this paper.

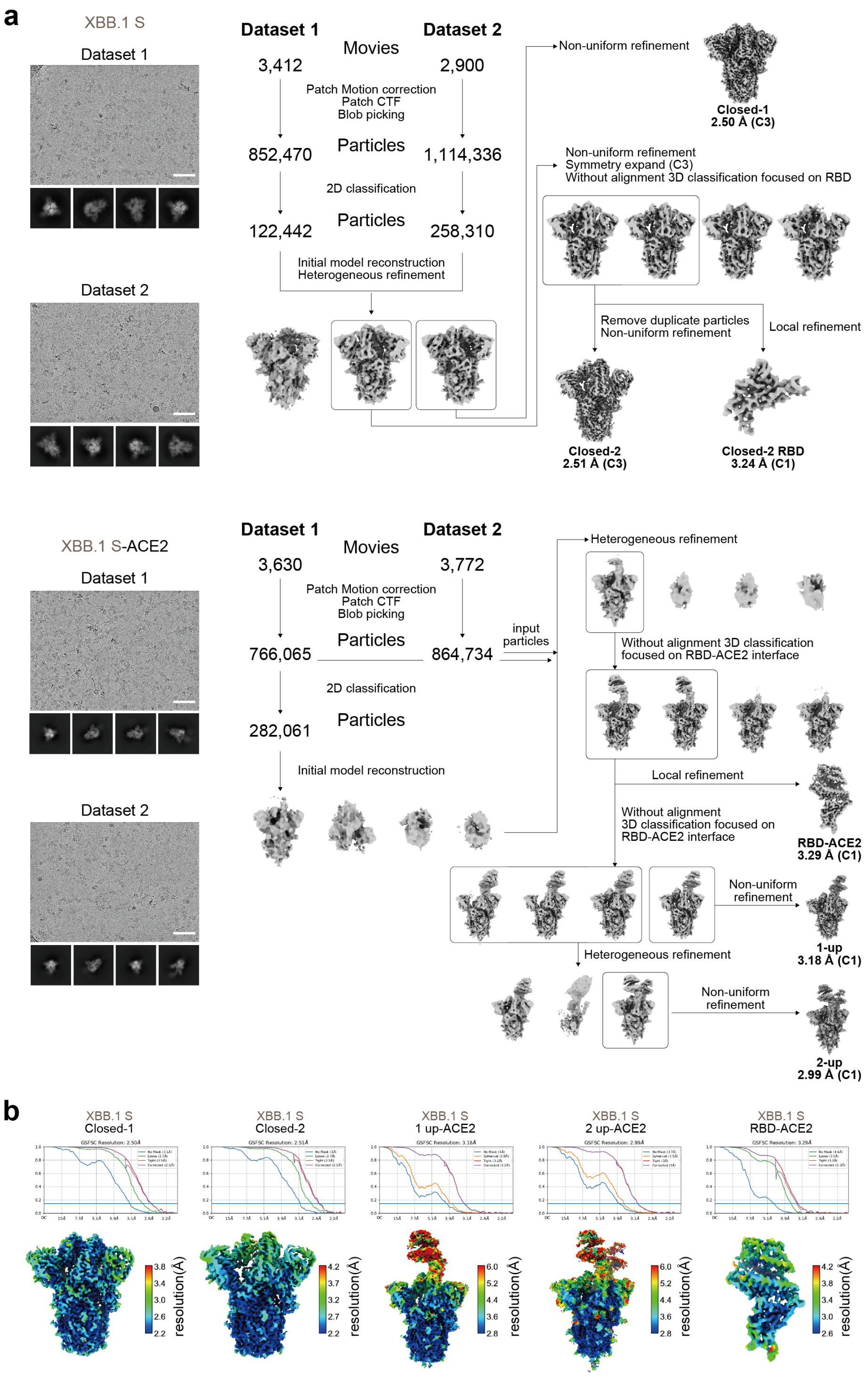

Supplementary Fig. 3. Workflow of cryo-EM data processing

a, (Left) Representative micrograph (scale bars, 50 nm) and 2D class images. (Right) Cryo-EM data processing flowchart for XBB.1 S and XBB.1 S–angiotensin converting enzyme 2 (ACE2).

b, Global resolution assessment of cryo-EM maps by gold-standard Fourier shell correlation (FSC) curves at the 0.143 criteria. Local resolution is colored blue to red at each range.

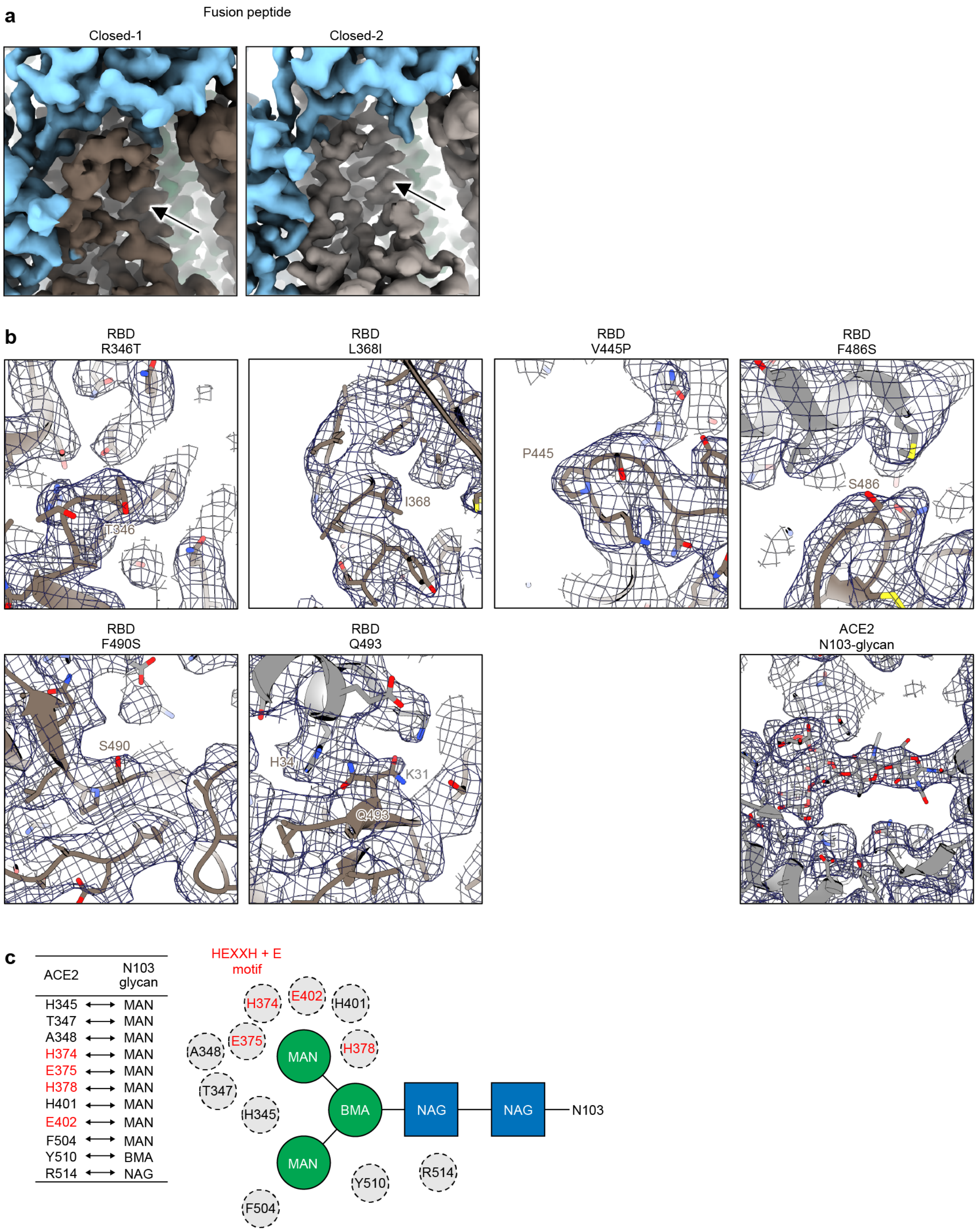

Supplementary Fig. 4. Cryo-EM maps and a schematic diagram of glycan-amino acid residue interactions.

a, Close-up view of cryo-EM maps around fusion peptide in the closed-1 or closed-2 state of XBB.1 spike (S) protein.

b, Cryo-EM maps of residues highlighted in **Fig. 4d**. The map is shown in blue mesh using the focused map on receptor binding domain (RBD)-angiotensin-converting enzyme 2 (ACE2) interface.

c, A list and a schematic illustration of ACE2 residues surrounding the N103-linked glycan of ACE2. Residues of the HEXXH motif are highlighted in red.

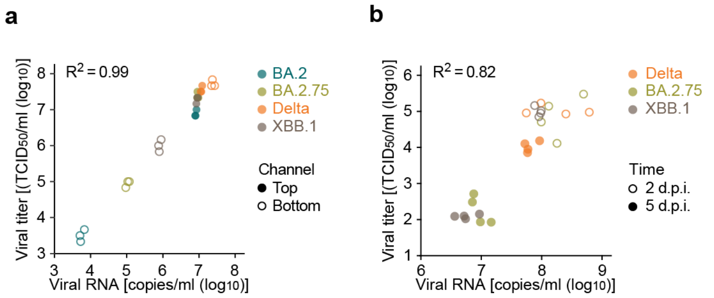

Supplementary Fig. 5. Correlation between the viral titer and viral RNA load
a, Clinical isolates of BA.2, BA.2.75, Delta and XBB.1 were inoculated into airway-on-a-chip. At 6 days post-infection (d.p.i), the culture supernatant was collected from both the top and bottom channels of an airway-on-a-chip. The copy numbers of viral RNA in the culture supernatant (shown in **Fig. 5h**) and viral infectious titer on VeroE6/TMPRSS2 cells [50% tissue culture infectious dose ($TCID_{50}$/ml)] of the collected culture supernatant were quantified. **b,** Syrian hamsters were intranasally inoculated with Delta, BA.2.75 and XBB.1. Four hamsters per group were euthanized at 2 and 5 d.p.i. and used for virological analysis. The viral RNA load (shown in **Fig. 6b**) and viral titer ($TCID_{50}$/ml) of lung periphery of infected hamsters (n=4 per infection group) were quantified. Source data are provided with this paper.

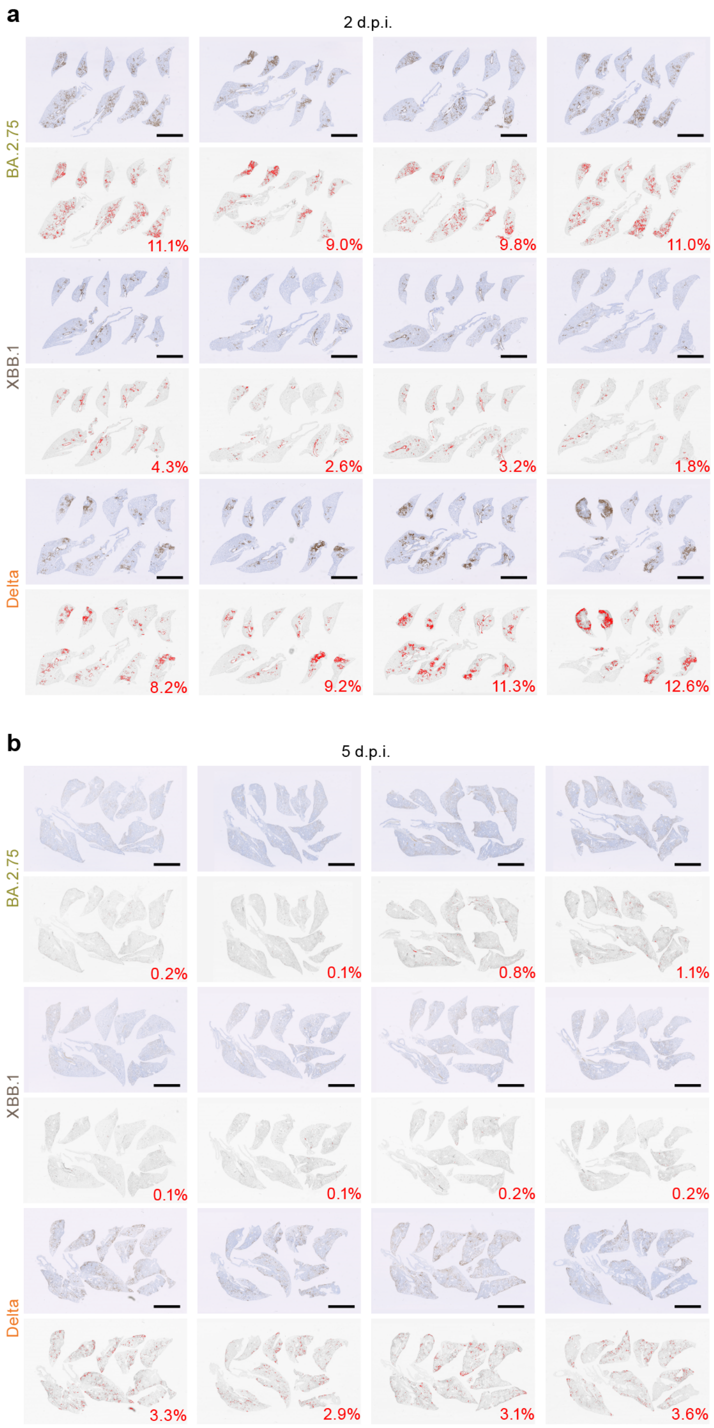

Supplementary Fig. 6. Histological observations in infected hamsters

Immunohistochemical (IHC) analysis of the SARS-CoV-2 nucleocapsid (N) protein in the lungs of infected hamsters at 2 days post-infection (d.p.i.) (**a**) and 5 d.p.i (**b**) (4 hamsters per infection group). In each panel, IHC staining (top) and the digitalized N-positive area (bottom, indicated in red) are shown. The red numbers in the bottom panels indicate the percentage of the N-positive area. Summarized data are shown in **Fig. 6c, right**. Scale bars, 5 mm.

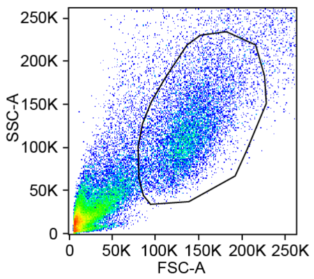

Supplementary Fig. 7. Gating strategy for flow cytometry

A representative dot plot of the gating for flow cytometry is shown. FSC, forward scatter; SSC, side scatter.
